# Polyanionic Drugs and Viral Oncogenesis: a Novel Approach to Control Infection, Tumor-associated Inflammation and Angiogenesis

**DOI:** 10.3390/molecules13112758

**Published:** 2008-11-06

**Authors:** Chiara Urbinati, Paola Chiodelli, Marco Rusnati

**Affiliations:** Unit of General Pathology and Immunology, Department of Biomedical Sciences and Biotechnology, School of Medicine, University of Brescia, Italy; E-mails: urbinati@med.unibs.it (C.U.); chiodell@med.unibs.it (P.C.).

**Keywords:** Angiogenesis, Cancer, Infectious diseases, Polyanionics

## Abstract

Polyanionic macromolecules are extremely abundant both in the extracellular environment and inside the cell, where they are readily accessible to many proteins for interactions that play a variety of biological roles. Among polyanions, heparin, heparan sulfate proteoglycans (HSPGs) and glycosphingolipids (GSLs) are widely distributed in biological fluids, at the cell membrane and inside the cell, where they are implicated in several physiological and/or pathological processes such as infectious diseases, angiogenesis and tumor growth. At a molecular level, these processes are mainly mediated by microbial proteins, cytokines and receptors that exert their functions by binding to HSPGs and/or GSLs, suggesting the possibility to use polyanionic antagonists as efficient drugs for the treatment of infectious diseases and cancer. Polysulfated (PS) or polysulfonated (PSN) compounds are a heterogeneous group of natural, semi-synthetic or synthetic molecules whose prototypes are heparin and suramin. Different structural features confer to PS/PSN compounds the capacity to bind and inhibit the biological activities of those same heparin-binding proteins implicated in infectious diseases and cancer. In this review we will discuss the state of the art and the possible future development of polyanionic drugs in the treatment of infectious diseases and cancer.

## Introduction

**Figure 1:**
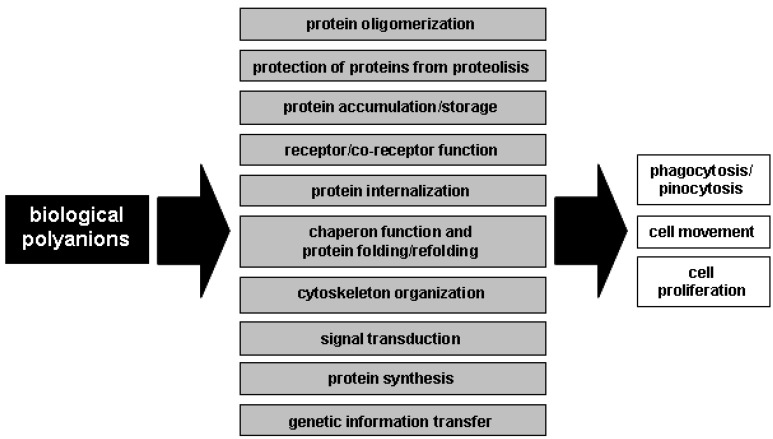
Interplay of the biological processes mediated by biologic polyanions.

Polyanionic macromolecules are extremely abundant both in the extracellular environment and inside the cell (Table 1), where they are readily accessible to proteins for interactions that regulate different physiological and pathological functions (Figure 1):

i) in biological fluids and at the cell surface, large polyanions such as glycosaminoglycans (GAGs) bring proteins together to favour protein-protein interactions [1].
ii) GAGs and heparan sulfate proteoglycans (HSPGs) of the extracellular matrix (ECM) act as a storage site for various proteins. They also protect bound proteins from degradation, prolong their lifespan and regulate their bioavailability [2].
iii) HSPGs and neuraminic acid (NeuAc)-bearing glycosphingolipids (GSLs) and glycoproteins present on the surface of eukaryotic cells act as coreceptors for various ligands [2,3] and even as direct signalling receptors [2]. Also, they mediate cell internalization of small proteins [4].
iv) these same cell-surface polyanions act as receptors for many human viruses [5], bacteria and protozoa [6], being thus implicated in the arise of various infectious diseases.
v) at an intracellular level, polyanions such as GAGs [7] and polyglutamate [8] are endowed with chaperone-like activity and/or the capacity to stabilize and even refold target proteins [9].
vi) the polyanionic nature of many intracellular second messengers plays a major role in their biology (i.e. inositol phosphate). Each event of phosphorylation results in a gain of two negative charges, and the degree of phosphorylation can be quite extensive [8]. This conveys to phosphorylated proteins a polyanionic feature with consequent “docking” properties that allow the binding and activation of other second messengers.
vii) the phosphorylated form of tubulin and actin (see above) and nucleic acids are intracellular polyanions that play essential roles in cytoskeleton organization, cell division, DNA transcription and protein synthesis.

In summary, polyanions are ubiquitous molecules (Table 1) involved in a wide array of important biological and pathological processes, inferring the possibility that polyanionic analogs endowed with agonist or antagonist potential can lead to the rescue of impaired biological processes or to the inhibition of pathological events. 

**Table 1:** Distribution of natural polyanions.

compartment	polyanions
**extracellular environment** **(biologic fluids, extracellular matrix)**	free GAGs and GSLs, proteoglycans
**cell membrane**	membrane-associated proteoglycans and GSLs, NeuAc-bearing glycoproteins
**intracellular environment** **(different compartments/organules)**	GAGs, proteoglycans, GSLs, RNA, DNA, ribosomes, phosphorylated proteins, actin, microtubules

NeuAc is a major eukaryotic cell surface anion whose expression is regulated by different cytokines [10]. It can be found associated to GSLs and to acidic glycoproteins such as integrins, both known modulators of microbial infection [6,11,12,13], angiogenesis and oncogenesis [14,15,16]. 

Heparin, GAGs and HSPGs are present in biological fluids, in ECMs, at the cell membrane and inside the cell, where they bind to hundreds of eukaryotic, prokaryotic and viral proteins [17,18]. As well as GSLs, they are involved in the modulation of microbial infection, angiogenesis and oncogenesis. Heparin, GAGs and HSPGs are far the best studied extracellular polyanions [19,20]. For these reasons, these molecules and their polysulfated (PS) or polysulfonated (PSN) antagonists will be the main subject of this review, not forgetting that the concepts that will be here discussed may be applied also to the other polyanions mentioned above (in particular GSLs).

**Figure 2:**
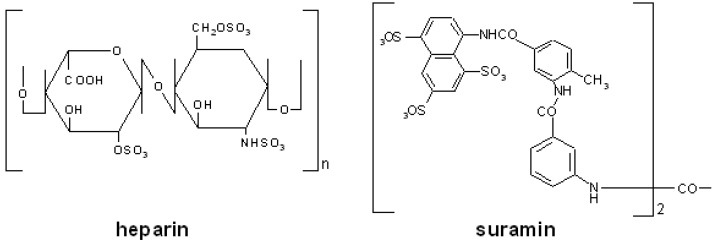
Chemical structure of the prototypic PS and PSN compounds heparin and suramin.

Heparin is a sulfated GAG produced by mast cells and mainly composed of regular trisulfated disaccharide sequences made up of alternating α-1,4-linked residues of 2-*O*-sulfated L-IdoA (IdoA2) and *N*-,6-*O*-disulfated GlcN (where IdoA is iduronic acid and GlcN is glucosamine). These regular sequences are occasionally interrupted by nonsulfated uronic acids (either GlcA or IdoA) (where GlcA is glucuronic acid) and by undersulfated hexosamines (GlcNS, GlcNAc, GlcNAc6S) (where Ac is acetate) (Figure 2). 3-*O*-sulfated glucosamines (GlcNS3S or GlcNS3S6S) are minor constituents of heparin but they are essential for the interaction with antithrombin III (ATIII) [21]. In turn, this interaction is essential for the anticoagulant activity of heparin. Accordingly, heparin and derivatives have long been used as anticoagulant/antithrombotic drugs [22].

Heparin binds also to a variety of biologically active polypeptides including growth factors, cytokines, and microbic proteins [23]. Accordingly, heparin-like prodrugs have been produced devoid of anticoagulant activity but endowed with the capacity to bind to distinct proteins for therapeutical intervention in a variety of diseases [20]. These modified heparins have been obtained mainly by selective desulfations, carboxyl reduction, replacement of *N*-sulfated groups with *N*-acetylated groups 

and chain fragmentation of native heparin purified from animal tissues. Alternatively, heparin-like molecules can be obtained by selective chemical sulfation of K5, an unsulfated polysaccharide from *Escherichia Coli* with the same structure of the heparin precursor *N*-acetyl heparosan [24].

Beside heparin, several other natural PS GAGs have been employed in a wide array of therapeutical applications [25]. Also, numerous PS plant compounds and marine products (often referred to as nutraceuticals) have been tested for their potential clinical applications [26]. Finally, a long list of PS compounds have been chemically synthesized (see below).

Suramin is a PSN naphthylurea mainly used for the treatment of trypanosomiasis [27] and onchocerciasis [28]. Suramin contains eight benzene rings, four of which are fused in pairs (naphthalene groups), four amide groups in addition to the one of urea and six sulfonated groups (Figure 2). Starting from suramin, several derivatives have been produced to be used for the treatment of several pathologies, including infectious diseases [29,30] and cancer [31].

## Polyanionic compounds and infectious diseases

**Figure 3:**
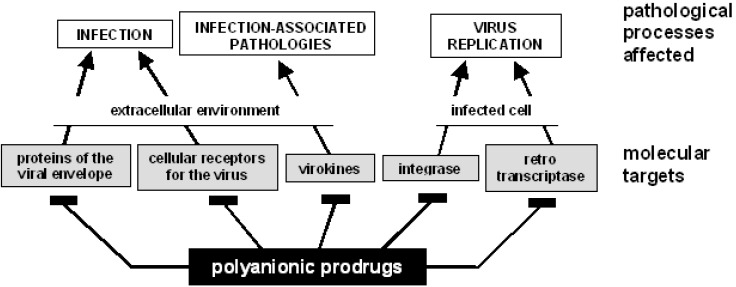
Polyanionic prodrugs affect different steps of the retroviral cycle.

Viruses rely on their hosts’ apparatus for gaining access to cells and to get support for replication and survival. HSPGs act as receptors for several viruses [32]. Accordingly, numerous PS/PSN exert antiviral activity by different mechanisms (Figure 3 and Table 2):

i) extracellularly, PS/PSN compete with HSPGs for the binding to the main determinants of virus infectivity such as the human immunodeficiency virus (HIV) gp120 glycoprotein [33,34,35,36]. The degree of sulfation as well as the disposition of sulfated groups of the saccharidic chain of HSPGs seems to be of particular importance for their capacity to interact with viral proteins. One of the better characterized case is that of herpes virus, whose glycoproytein gD needs to bind specifically to 3-*O*-sulfated glucosamines to allow virus binding and entry into target cells [37].
ii) alternatively, PS/PSN bind and mask entry receptors for viruses such as the HIV receptor CD4 [38].
iii) PS/PSN enter the cell and prevent virus replication by inhibiting viral enzymes (such as reverse transcriptase [39,40,41], integrase [42] or the RNAse [43] of HIV), or viral transactivating factors (such as the transactivating factor Tat of HIV [44]).
iv) natural PS and nutraceuticals enhance inflammatory and immunitary responses to viruses and bacteria with still unknown mechanisms. Heparin and heparan sulfate (HS) increase cytotoxic T lymphocytes responses and production of cytokines [45]. Sulfatides trigger TNF-α and CXCL8 overexpression in neutrophils [46]. Sulfated polysaccharides from *Grifola frondosa* [47] increase proliferation and tumoricidal activity of lymphocytes and macrophages. In these latter cells, exopolysaccharide from marine microalga *Gyrodinium impudicum* [48] increases phagocytosis, lysosomal enzyme activity, production of nitrite, H_2_O_2_, TNF-α and IL-6.
v) virokines are virally encoded proteins secreted from infected cells that modulate different aspects of the host immune system to maintain a suitable habitat for viral replication. In addition, they often act as cytokines that contribute to cell proliferation [49]. Myxoma virus CC-chemokine inhibitor (M-T1) is a poxvirus secreted virulence factor that binds to sulfated GAGs of target cells affecting chemokines function [50], while the E163 protein from Ectromelia virus binds to the GAGs binding site of CXCL10 and CXCL12, thus inhibiting their interaction with HSPGs and consequent biological activities [51]. These examples underline the interplay existing among viral proteins, chemokines and GAGs pointing to its relevance as a target for the development of PS compounds with therapeutical value. HIV Tat can be released by infected cells, acting as a virokine that binds to HSPGs [52] and stimulates different HIV-non permissive cells, contributing to AIDS-associated pathologies such as central and peripheral neuropathies, immune suppression and tumorigenesis [44,53]. Several PS/PSN effectively bind and sequester Tat in the extracellular environment, preventing its interaction with target cells and inhibiting some of its pathological effects [44].

Although GAGs and HSPGs have been studied mainly for their role as entry receptors for viruses, they are also involved in bacterial and protozoan diseases [6]. Accordingly, several PS/PSN have been demonstrated to inhibit infection by these microrganisms (Table 3).

On the other hand, although HSPGs are the most studied polyanions of the surface of eukaryotic cells, also GLSs play an important role as receptors for viruses, bacteria and related toxins (see above).

**Table 2:** PS/PSN that inhibit infections by viruses.

PS compounds	target virus
**unmodified/ chemically modified heparin/HS**	**DNA:** HSV [54,55], CMV [56], FMDV [57], HBV [58], HCV [59], HPV [60], HHV-7 [61], HHV-8 [62], VV [63], VZV [64] **RNA**: HTLV [65], HIV, VSV, Sindbis [63,66], DENV, JEV [67], Tacaribe, Junin [68], RSV, influenza A [69,70]
**chondroitin sulfate**	**DNA:** HIV [71]
**carrageenans**	**DNA:** HSV, CMV, HPV [72], VV [63] **RNA**: HIV, Sindbis, VSV [63], DENV [73], HAV [74], CHIKV, SFV [75], Tacaribe, Junin [68]
**xylomannan sulfate F6**	**DNA:** HSV, CMV [42] **RNA**: HIV, influenza A/B, Tacaribe, Junin [42]
**galactan sulfate**	**DNA:** HSV, CMV, VV [42] **RNA**: HIV, Sindbis, SFV, VSV, influenza A, RSV [42], DENV [76]
**fucoidan**	**DNA:** HSV, CMV [63] **RNA**: HIV, Sindbis, VSV [63], RSV [77], SFSV [78], CHIKV, SFV [75], Tacaribe, Junin [68], HTLV [79]
**rhamnan sulfate**	**DNA:** HSV, CMV [80] **RNA**: HIV [80]
**cellulose sulfate**	**DNA:** HSV [81], HPV [82] **RNA**: HIV [83]
**dextran sulfate**	**DNA:** HSV, CMV, HPV, VV [82], HBV [58], HHV-7 [61] **RNA**: HIV , Sindbis, VSV [63], RSV, influenza A, Tacaribe, Junin, SFV [42], CHIKV [75], SFSV [78], YFV [84], RV [85] HTLV [86]
**colominic acid**	**RNA**: HIV [87], RTV [88]
**curdlan sulfate**	**DNA:** CMV [89] **RNA**: HIV [89]
**glyloid sulfate 4324**	**RNA**: RV [85]
**PI 88**	**DNA:** HSV [54] **RNA**: DENV, JEV [67]
**K5 derivatives**	**DNA:** HPV [60] **RNA**: HIV [90]
**PPS**	**DNA:** HSV, CMV, HHV-7 [61], VV [42] **RNA**: HIV, Sindbis, VSV [63], RSV, influenza A, Tacaribe, Junin [42], SFSV [78], DENV, JEV [67]
**polyester**	**RNA**: HIV [91]
**chitin derivatives**	**DNA:** HSV [92]
**Y-ART-4**	**RNA**: HIV [93]
**PSN compound**	**target virus**
**suramin**	**DNA:** HBV [94], HCV [95], HHV-8 [96], HSV [97] **RNA**: HTLV-1 [98]; HIV [99]
**suramin analogs**	**DNA:** CMV [100] **RNA**: HIV [101]
**PSS**	**DNA:** HTLV [98], HSV [81], HBV [58], HHV-7 [61], HPV [82], CMV [100] **RNA**: HIV [102], RSV, influenza A [69], YFV [84]
**porphyrins**	**RNA**: HIV [103]

PI 88, phosphomanno pentaose sulfate; Y-ART-4, nonatyrosine N- and O-1-9-decasulfate; PPS, pentosan polysulfate; PSS, poly(sodium 4-styrene sulfonate); CHIKV, Chikungunya virus; CMV, cytomegalovirus; DENV, Dengue virus; FMDV, foot-and-mouth disease virus; HAV, hepatitis A virus; HBV, hepatitis B virus; HCV, hepatitis C virus; HHV, human herpes virus; HPV, human papilloma virus; HSV, herpes simplex virus; HTLV, human T-cell leukemia virus; JEV, Japanese encephalitis virus; RSV, respiratory syncytial virus; RTV, rotavirus; RV, rubella virus; SFSV, sanfly fever sicilian virus; SFV, Semliki forest virus; VSV, vescicular stomatitis virus; VV, vaccinia virus; VZV, varicella zoster virus; YFV; yellow fever virus.

**Table 3:** PS/PSN compounds that inhibit infections by bacteria and protozoa.

PS compounds	target microrganism
**unmodified and chemically modified heparin/HS**	**bacteria** *: Staphylococcus epidermidis* [104], *Staphylococcus aureus* [105], *Staphylococcus hemolyticus* [106], *Listeria monocitogenes* [107], *Helicobacter pylori* [108], *Escherichia coli* [109], *Borrelia burgdorferi* [110], *Neisseria gonorroheae, Chlamidia trachomatis* [111], *Mycobacterium tubercolosis* [112], *Burkholderia pseudomallei* [113], *Legionella pneumophila* [114] **protozoa**: *Leishmania amazonensis* [115], *Trypanosoma brucei* [116], *Plasmodium falciparum* [117,118], *Tripanosoma cruzi* [119], *Toxoplasma gondii* [120], *Giardia lamblia* [121]
**chondroitin sulfate**	**bacteria** *: Staphylococcus epidermidis*, *aureus, hemolyticus* [106], *Listeria monocitogenes* [107] **protozoa**: *Plasmodium falciparum* [122], *Toxoplasma gondii* [120]
**carrageenans**	**bacteria** *: Helicobacter pylori* [108] **protozoa**: *Plasmodium falciparum* [123]
**fucoidan**	**bacteria** *: Staphylococcus epidermidis, aureus, hemolyticus* [106], *Anaplasma phagocytophilum* [124], *Helicobacter pylori* [125] **protozoa**: *Cryptosporidium parvum* [126], *Toxoplasma gondii* [127]
**cellulose sulfate**	**bacteria** *: Gardenella vaginalis* [128] **protozoa**: *Plasmodium falciparum, Toxoplasma gondii* [127]
**dextran sulfate**	**bacteria** *: Staphylococcus epidermidis, aureus, hemolyticus* [106], *Helicobacter pylori* [108], *Borrelia burgdorferi* [110], *Neisseria gonorrhoeae, Chlamidia trachomatis* [111], *Bacillus anthracis* [113], *Legionella pneumophila* [114] **protozoa**: *Plasmodium falciparum* [129]
**PI 88**	**protozoa**: *Plasmodium falciparum* [129]
**PPS**	**bacteria** *: Staphylococcus epidermidis, aureus, hemolyticus* [106], *Neisseria gonorrhoeae, Chlamidia trachomatis* [111] **protozoa**: *Plasmodium falciparum* [129]
**PSN compounds**	**target microrganism**
**suramin**	**protozoa**: *Plasmodium falciparum* [130], *Trypanosoma cruzi* [131]
**suramin analogs**	**protozoa**: *Plasmodium falciparum* [130]
**PSS**	**bacteria** *: Neisseria ghonorrhoeae, Chlamydia trachomatis* [132], *Gardenella vaginalis* [128]

Accordingly, lectins (that bind and mask cell surface-associated GLSs) and exogenous GLSs (that compete with cellular GLSs for the binding to microrganisms) have been taken in consideration as pathogens inhibitors [13,133]. 

## Polyanionic compounds, tumor and angiogenesis 

**Figure 4:**
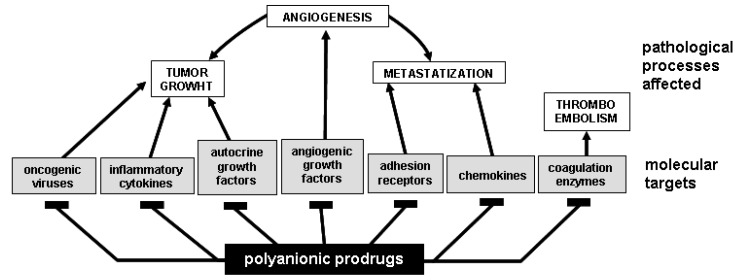
Polyanionic prodrugs affect different steps of cancerogenesis.

PS/PSN exert significant anti-tumor effects *in vitro* and even in clinical trials [134] by different mechanisms (Figure 4): 

i) oncogenic viruses are involved in the arise of human malignancies such as carcinomas of the cervix uteri, hepatocellular carcinomas and lymphomas [135]. Thus, dealing with viral oncogenesis, PS/PSN can exert a so early effect as to block cell infection and transformation, thus preventing the arise of the tumor.
ii) PS/PSN can be used for the prevention of thromboembolic diseases that significantly contribute to the morbidity and mortality of oncological patients [136].
iii) different natural PS compounds exert an anti-tumor activity acting directly on tumor cells. Heparin, HS, dextran sulfate and fucoidan induce apoptosis of hepatoma and lymphoma cells [137,138]. Various PS polysaccharides inhibit tumour metastasis by blocking tumour-derived glycosidases and proteases such as heparanases [139] and matrix metallo proteinases [140].
iv) several natural PS/PSN stimulate an antitumoral immune response [47], although the mechanism(s) by which they exert this effect is not fully elucidated.
v) chronic inflammation promotes tumor progression mainly because pro-inflammatory cytokines suppress apoptosis and stimulate cell proliferation, angiogenesis, invasion, and metastasis [141]. Interestingly, many of these tumor-promoting cytokines are heparin-binding proteins [18] that can be intercepted and blocked by PS/PSN prodrugs.
vi) tyrosine kinases (TKs) are intracellular signal transducing enzymes implicated in tumor progression. Efforts have been made to develop antagonists that interfere with the interaction of TKs with their substrates. Small synthetic cell permeable PS/PSN molecules can be internalized by cells [41], suggesting their use as intracellular antagonists of cytoplasmic TKs involved in oncogenesis.
vii) angiogenesis, the process of new blood vessel formation from pre-existing ones, is an absolute requirement for tumor growth and metastatization [142]. Angiogenesis is mediated by angiogenic growth factors (AGFs) (Table 4) that stimulate an uncontrolled endothelial cell (EC) activation by interacting with specific TK receptors expressed on the EC surface [143]. However, to exert a full angiogenic response, some AGFs must interact also with EC surface NeuAc-bearing gangliosides [3,18] and HSPGs [18]. In effect, almost all the AGFs are heparin-binding proteins and may as well bind to GLSs [18]. A wide array of PS/PSN exert a potent antiangiogenic effect *in vitro* and *in vivo* by binding and sequestering AGFs in the extracellular environment, thus preventing their action on ECs (Table 4). The same effect can be exerted by free GLSs [18,144,145].

**Table 4:** PS/PSN compounds that bind AGFs and inhibit pro-angiogenic biological activities *in vitro* and/or angiogenesis *in vivo.*

polysulfated compounds	target AGF
**unmodified and chemically modified heparin/HS**	VEGF [146,147,148,149], FGF2 [2,150,151,152], HGF, PDGF [153,154,155], Tat [1,156,157], midkine [158], angiogenin [159], angiopoietin [160], pleiotrophin [161]
**chondroitin sulfate**	FGF2 [150], PDGF [162], midkine [158], peliotrophin [161]
**oligosaccharides from alginic acid of seaweed**	VEGF [163]
**polysaccharides from *Antrodia cinnamomea***	VEGF [154]
**carrageenans**	FGF2 [164,165]
**fucoidan**	VEGF, FGF2 [166,167]
**SargA (from *Sargassum Stenofillum)***	FGF2 [168]
**dermatan sulfate**	FGF2 [150], HGF [169]
**laminarin sulfate**	FGF2 [170]
**SPMG**	FGF2 [171], Tat [172]
**sulfatides**	FGF2 [144], HGF [173], midkine [174]
**dextran sulfate**	VEGF, FGF2 [175,176], HGF [177]
**exopolysaccharide from *Alteromonas infernos***	VEGF, FGF2 [178]
**heparin-carrying polystyrene**	VEGF, FGF2, HGF [179]
**heparin oligomer glycodendrimers**	FGF2 [180]
**heparin-mimicking sulfated peptides**	VEGF [181]
**suleparoide (HS analog)**	FGF2 [182]
**K5 derivatives**	FGF2 [183,184], Tat [185]
**PI-88 and analogs**	VEGF, FGF2 [186,187]
**RGTAs (synthetic GAG)**	VEGF [188], FGF2 [189]
**sucrose octasulfate**	FGF2 [190]
**beta-(1->4)-galacto oligosaccharides**	FGF2 [191]
**PPS**	FGF2 [192], Tat [193]
**β-cyclodextrin**	FGF2 [194], Tat [195]
**PSN compounds**	**target AGF**
**suramin**	VEGF [196], FGF2 [197,198]
**suramin analogs**	VEGF, PDGF [199], FGF2 [200], Tat [29,201]
**PSS**	FGF2 [202], Tat [203]

The use of PS/PSN antiangiogenic drugs for the treatment of cancer has some advantages: AGFs such as FGFs and hepatocyte growth factor (HGF) act as pleiotropic cytokines that, in addition to neovascularization, induce proliferation of tumor cells. Targeting these pleiotropic cytokines with PS/PSN may thus gain benefits not only from the inhibition of neovascularization but also from the direct inhibition of tumor cell proliferation (Figure 4).

## Polyanionic compounds as drugs: drawbacks and perspectives

A tight correlation exists between infectious diseases and tumors:

i) some viruses are endowed with a well known transforming capability, while some bacterial infections are known to favour the arise of tumors.
ii) some virokines and bacterial toxins play a role in the development of tumors.
iii) infectious diseases trigger inflammation that, in turn, triggers neovascularization, a process that is an absolute requirement for tumor growth and metastatization.

Amazingly, infection, angiogenesis and tumor growth are mediated by viral proteins, cytokines, chemokines and proteases that often share the need to bind to polyanionic structures of the cell (mainly HSPGs and GLSs) to exert their pathological effects. These interactions can be considered as targets for the development of novel polyanionic drugs for the treatment of infectious diseases and cancer. In effect, several PS/PSN (Table 2, Table 3 and Table 4) and sialylglycoconjugates [18,144,145,204] have been demonstrated to exert anti-microbial, anti-angiogenic and anti-tumor activity.

The use of PS/PSN as drugs is limited by two important drawbacks: their (possible) anticoagulant activity and their aspecificity, both the properties mainly relying on the capacity of PS/PSN compounds to interact simultaneously with coagulation enzymes and other heparin-binding proteins. 

As already mentioned, the anticoagulant activity of heparin depends on a structurally defined ATIII-binding pentasaccharide where the 3-*O*-sulfate group at residue 3 is the key residue [21]. This knowledge allowed the successful production of several PS/PSN devoid of anticoagulant activity but retaining their capacity to bind and neutralize different cytokines and growth factors.

On the other hand, the tendency of PS/PSN to bind aspecifically to different heparin-binding proteins started a series of ambitious studies aimed at the characterization of the molecular bases of each distinct HS/protein interaction. This with the equally ambitious goal to produce PS/PSN specifically directed against a single target. Rather than reach their goals, these studies showed that HS/protein interactions depend more on the overall degree of sulfation of HS than on their fine structure [205], making unlike the possibility to produce PS/PSN endowed with a tight specificity. However, the capacity of PS/PSN to bind different proteins simultaneously may represent an advantage rather than a drawback. In effect, a certain degree of aspecificity may increase the therapeutical efficacy of a PS/PSN compound in selected pathological settings:

i) PS/PSN such as PPS [40,193,206,207,208], suramin and analogs [29,209,210] and synthetic sulfonic acid polymers [203,211,212] are able to simultaneously bind and mask gp120 (thus inhibiting HIV infection), neutralize intracellular enzymes (such as reverse transcriptase and integrase) and inhibit the extracellular form of Tat (implicated in several AIDS-associated pathologies [213]). These observations suggest the possibility (and the opportunity) to design and produce polyanionic drugs able to bind different viral proteins simultaneously, thus interfering at once with different steps of the virus cycle (Figure 3). This “multivalent” binding capacity may limit the arise of drug resistant viral strains that, to date, represents the major limit of common antiviral therapies aimed to a single molecular target.
ii) in advanced stages of human tumors, usually characterized by a high degree of vascularization, different AGFs are expressed at high levels at the same time, suggesting that tumor neovascularization is often the result of the simultaneous action of different AGFs [214]. Thus, the possibility to efficiently inhibit neovascularization *in vivo* by using an inhibitor specifically directed against a single AGF is far-off [214], while “multivalent” polyanionic drugs (able to bind different AGFs) may be more effective in inhibiting angiogenesis and consequent tumor progression *in vivo.*

As described in introduction and in Table 1, beside heparin/HSPGs, many other polyanions exists that play important physiological roles. At this regard, it has been inferred that, being protein/polyanion interactions of electrostatic nature, a protein endowed with the capacity to bind to a given polyanion might as well bind to others [8]. In effect, FGF2, Tat, HGF, CXCL8, midkine and platelet derived growth factor (PDGF) bind to both heparin/HSPGs and negatively charged NeuAc residues present on GSLs [18] and/or on integrins [M. Rusnati, unpublished observations]. Nevertheless, free gangliosides bearing NeuAc residues inhibit the binding of FGF2 to cell-associated gangliosides without affecting that to HSPGs [144]. Also, selected sulfated K5 derivatives inhibit the binding of HIV-Tat to HSPGs without affecting the binding to integrins [185]. These data indicate that, although polycationic proteins can bind simultaneously to different cellular polyanions, it is possible to produce synthetic polyanionic antagonists able to “discriminate” among the various interactions.

It must be pointed out however that polyanionic compounds able to prevent the interaction of a given ligand with different receptors can be an advantage in different situations:

i) for the prevention of infection by viruses such as HIV, influenza virus and RSV that need to interact with both HSPGs (see above) and gangliosides [204,215,216] for their entry in host cells.
ii) for the inhibition of angiogenesis and tumor growth driven by those AGFs that need to interact with both HSPGs and GLSs to exert a full angiogenic activity [3,18,144,145].

In effect, several functional similarities exist between GLSs and HSPGs that make them an ideal common target for polyanionic drugs (Table 5).

**Table 5:** Features shared by polyanionic HSPGs and GSLs.

Feature	HSPGs	GSLs
capacity to bind multiple proteins (via their negatively charged carboxyl or sulfated groups, respectively)	[18]	[18]
protection of bound proteins from proteolitic degradation	[2]	[144]
receptor/coreceptor function for AGFs and tumor growth factors (when cell membrane associated)	[2]	[3]
entry receptor for viruses (when cell membrane associated)	[32]	[6]
mobilization/shedding from cell membrane in the body fluids	[15]	[2]
antagonist activity (when in their soluble form)	[2]	[144]
chaperone function (when in their intracellular form)	[7]	[217]

**Figure 5:**
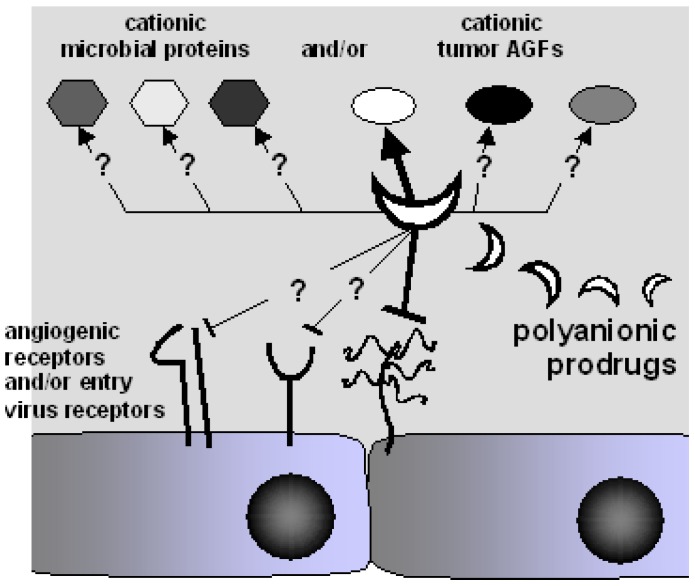
“Multivalent” binding capacity of polyanionic prodrugs.

In conclusion, the development of efficacious polyanionic anti-viral and/or anti-tumor agents depends on an appropriate balance between their specificity and their “multivalent” capacity to bind different pathological ligands and/or to compete with different cellular receptors (Figure 5). 

A polyanionic prodrug can bind specifically to one ligand hampering its binding to a specific receptor. Other polyanionic prodrugs bind instead different ligands hampering their interaction with multiple receptors. Depending on the physiopathological setting, an appropriate balance between specificity and “multivalent” binding can lead to efficient polyanionic drugs at the crossroad of tumor and infectious diseases.

The possibility to develop “multivalent” polyanionic drugs calls for systematic studies involving multiple target proteins and libraries of polyanionic compounds as large as possible. In this wiev, the use of automated oligosaccharide synthesizer [218] and/or carbohydrate microarrays [219] is mandatory. The feasibility of this approach is sustained by “pilot” studies performed with libraries of heparin-derived octasaccharides [220], sulfated linked cyclitols [221], sulfated K5 polysaccharides [222], suramin-like PSN distamycin derivatives [29] and HS-mimetic glycoconjugates [223]. 

Important contributions to the development of “polyanionic-based” anti-viral and/or anti-tumor therapies should be given also by *in silico* screening of protein/polyanion interactions based on molecular dynamics simulation of the docking events between the binding partners [224] and/or by NMR studies aimed to identify the conformational features required to polyanions and proteins to bind each other [225]. In this way, the “fishing” approach implicit in the screening of large libraries would be integrated to “rational design” studies, increasing the possibility to successfully produce potent “multivalent” drugs acting at the crossroad of tumor and infectious diseases.
